# Investigating the Causal Effect of Brain Expression of *CCL2*, *NFKB1*, *MAPK14*, *TNFRSF1A*, *CXCL10* Genes on Multiple Sclerosis: A Two-Sample Mendelian Randomization Approach

**DOI:** 10.3389/fbioe.2020.00397

**Published:** 2020-05-05

**Authors:** Teresa Fazia, Andrea Nova, Davide Gentilini, Ashley Beecham, Marialuisa Piras, Valeria Saddi, Anna Ticca, Pierpaolo Bitti, Jacob L. McCauley, Carlo Berzuini, Luisa Bernardinelli

**Affiliations:** ^1^Department of Brain and Behavioural Sciences, University of Pavia, Pavia, Italy; ^2^Bioinformatics and Statistical Genomics Unit, Istituto Auxologico Italiano IRCCS, Milan, Italy; ^3^Molecular Biology Laboratory, Istituto Auxologico Italiano IRCCS, Milan, Italy; ^4^John P. Hussman Institute for Human Genomics, Miller School of Medicine, University of Miami, Miami, FL, United States; ^5^Dr. John T. Macdonald Foundation Department of Human Genetics, Miller School of Medicine, Miami, FL, United States; ^6^Divisione di Neurologia, Presidio Ospedaliero S. Francesco, ASL Numero 3 Nuoro, Nuoro, Italy; ^7^Centro di Tipizzazione Tissutale, S.I.T., Presidio Ospedaliero S. Francesco, ASL Numero 3 Nuoro, Nuoro, Italy; ^8^Centre for Biostatistics, University of Manchester, Manchester, United Kingdom

**Keywords:** Multiple Sclerosis, Mendelian randomization, gene expression, family data, NF-κB signaling pathway

## Abstract

Multiple Sclerosis (MS) exhibits considerable heterogeneity in phenotypic expression, course, prognosis and response to therapy. This suggests this disease involves multiple, as yet poorly understood, causal mechanisms. In this work we assessed the possible causal link between gene expression level of five selected genes related to the pro-inflammatory NF-κB signaling pathway (i.e., *CCL2*, *NFKB1*, *MAPK14*, *TNFRSF1A*, *CXCL10*) in ten different brain tissues (i.e., cerebellum, frontal cortex, hippocampus, medulla, occipital cortex, putamen, substantia nigra, thalamus, temporal cortex and intralobular white matter) and MS. We adopted a two-stage Mendelian Randomization (MR) approach for the estimation of the causal effects of interest, based on summary-level data from 20 multiplex Sardinian families and data provided by the United Kingdom Brain Expression Consortium (UKBEC). Through Radial-MR and Cochrane’s Q statistics we identified and removed genetic variants which are most likely to be invalid instruments. To estimate the total causal effect, univariable MR was carried out separately for each gene and brain region. We used Inverse-Variance Weighted estimator (IVW) as main analysis and MR-Egger Regression estimator (MR-ER) and Weighted Median Estimator (WME) as sensitivity analysis. As these genes belong to the same pathway and thus they can be closely related, we also estimated their direct causal effects by applying IVW and MR-ER within a multivariable MR (MVMR) approach using set of genetic instruments specific and common (composite) to each multiple exposures represented by the expression of the candidate genes. Univariate MR analysis showed a significant positive total causal effect for *CCL2* and *NFKB1* respectively in medulla and cerebellum. MVMR showed a direct positive causal effect for *NFKB1* and *TNFRSF1A*, and a direct negative causal effect for *CCL2* in cerebellum; while in medulla we observed a direct positive causal effect for *CCL2*. Since in general we observed a different magnitude for the gene specific causal effect we hypothesize that in cerebellum and medulla the effect of each gene expression is direct but also mediated by the others. These results confirm the importance of the involvement of NF-κB signaling pathway in brain tissue for the development of the disease and improve our understanding in the pathogenesis of MS.

## Introduction

Multiple Sclerosis (MS) is a multifactorial disease with progressive neurodegeneration characterized by chronic inflammation and demyelination in the Central Nervous System (CNS) (Nylander and Hafler, 2012).

Many molecular events contribute to MS susceptibility and all these events are widely distributed across the many different cellular components of both the innate and adaptive immune system. Oligodendrocytes, the myelinating cells of the CNS, and neurons are mainly targeted by this wave of inflammation, that leads to cell death, that is closely associated with the manifestation of clinical symptoms (Mc Guire et al., 2013; Ghasemi et al., 2017). MS pathogenesis has both genetic and environmental factors. Research on genetic susceptibility to MS has been fueled by recent genome-wide association studies (GWASs), fine-mapping and meta-analysis (Hafler et al., 2007; International Multiple Sclerosis Genetics, 2011; International Multiple Sclerosis Genetics Consortium, 2019; Patsopoulos et al., 2011, 2013; International Multiple Sclerosis Genetics Consortium, Beecham et al., 2013; Andlauer et al., 2016; George et al., 2016). These studies have also highlighted loci never previously implicated in MS, which represents an opportunity to generate novel insight into the biological pathways involved in the disease. Most of the GWAS-highlighted loci that appear to operate via gene expression regulation, rather than protein coding, spurred the researchers to perform expression quantitative trait loci (eQTL) analyses that highlighted genes whose expression in specific tissues is regulated by loci associated with the disease, thus showing the potential functional consequences of certain MS associated variant (Edwards et al., 2013; Gibson et al., 2015).

The fact that other GWAS signals fall within specific signaling cascades, suggests that the understanding of how single variants with small odds ratio act in disease susceptibility could lie in the alteration in pathways, rather than in individual genes (Housley et al., 2015). More specifically, a GWAS conducted by International Multiple Sclerosis Genetic Consortium (IMSGC) in 2013 (International Multiple Sclerosis Genetics Consortium, Beecham et al., 2013) reported the nuclear factor kappa-light-chain-enhancer of activated B cells (NF-κB) pathway as significant in MS pathology.

NF-κB acts on many immune cells, and its constitutive activation leads to an increase inflammation in inflammatory and autoimmune diseases, such as MS (Yan and Greer, 2008). It is crucial for B and T lymphocytes’ development and proliferation, for production of pro-inflammatory cytokines, inflammatory mediators by dendritic cells, and neurotoxic mediators in microglia and astrocytes (Leibowitz and Yan, 2016). Many studies have reported the activation of NF-κB in the brain tissue of MS patients (Gveric et al., 1998; Bonetti et al., 1999; Lock et al., 2002). Approximately 20% of genome-wide MS susceptibility variants fall within and/or proximal to NF-κB signaling genes, including *NFKB1* or p105/p50 and *TNFRSF1A* (*TNFR1*), which cause decreased expression of the negative regulators of NF-κB (De Jager et al., 2009; Housley et al., 2015). As shown in a GWAS noise reduction (GWAS-NR), an approach to detect novel associations beyond those detected by traditional GWAS, variants regulating the activation and proliferation of immune effector cells, comprising key regulators of NF-κB signaling, are involved in the genetic susceptibility to MS (Hussman et al., 2016).

The available biological evidence led us to here focus on *a priori* selected set of genes, *CCL2*, *NFKB1*, *MAPK14*, *TNFRSF1A*, *CXCL10*, on the basis of their involvement in MS risk and in NF-κB signaling pathway. Should we conclude that these genes cause the disease through changes in their expression? To assess the possible causal links between their expression levels in ten different brain tissues [i.e., cerebellum (CRBL), frontal cortex (FCTX), hippocampus (HIPP), medulla (MEDU), occipital cortex (OCTX), putamen (PUTM), substantia nigra (SNIG), thalamus (THAL), temporal cortex (TCTX) and intralobular white matter (WHMT)] and MS we adopted a two-sample Mendelian Randomization (MR) approach. MR uses measured variation in DNA sequence, *Z*, to assess the possible causal effect of a biological phenotype, *X*, on a response variable, *Y* (Davey Smith and Ebrahim, 2003; Davey Smith and Hemani, 2014; Burgess et al., 2017), without requiring any experimental intervention on *X* to assess causality. The variable *Z* is said, in this case, to be an instrument for the estimation of the effect of interest. Ideally, to be a valid instrumental variable (IV) the MR approach requires the instrument, *Z*, to satisfy three validity conditions, precisely to be: (*i.*) associated (not necessarily in a causal way) with *X*; (*ii.*) independent of *Y* conditional on *X* and on the confounders (*U*) of the relationship between *X* and *Y* (no pleiotropy); and (*iii.*) furthermore independent of those confounders.

So far MR have been mainly used to investigate the causal effect of a high-level phenotype (e.g., *X* = body mass) on a specific medical outcome. Here we shift the causal exploration to the level of individual genes, which we do by letting *X* represent the level of expression in brain tissues of a specific gene and by letting the instrument *Z* consists of a collection of Single Nucleotide Polymorphisms (SNPs) that regulate the expression of that gene. In this way, thanks to MR, we may use the information provided by genotyping to address the question whether changes in the level of expression of the gene of interest represent causal influences on the disease, in the hope that this will then point to biological pathways of pathogenic relevance.

In this study the associations between the genetic variants and the disease and between the same genetic variants and the exposure were estimated respectively in two independent and non-overlapping samples: Sardinian multiplex families (Fazia et al., 2017) and data provided by the United Kingdom Brain Expression Consortium (UKBEC) (Trabzuni et al., 2011; Ramasamy et al., 2014).

In this application we are also interested to estimate the direct effect of multiple gene expressions belonging to the same NF-κB signaling pathway because it is plausible to believe that they are closely related. For the sake of argument, if we consider the expression of *NFKB1* as the exposure of major interest, we may want also to investigate if, beyond its direct effect, the effect of the genes belonging to the same pathway may mediate the relationship between *NFKB1* expression and disease. In other words, we want to estimate the direct effect of *NFKB1*. For this reason, in addition to the estimation of total causal effect via univariable MR, we also applied a multivariable MR (MVMR) in which a set of genetic instruments is used to predict a set of exposure variables, which are the expression levels in the different brain regions of the genes belonging to the same pathway, for the estimation of their direct effect. The same argument applies to all the genes in the pathway.

Thus, our aim here is to improve our understanding of the pathogenesis of MS, which is yet to be clarified, pointing to important candidate genes, in specific brain tissues, to be prioritized for further studies and potentially drug discovery.

## Materials and Methods

### Data Sources

The required instrument-outcome (i-o) associations and instrument-exposure (i-e) associations were provided by two separate and independent datasets.

(i) *Dataset 1* (*Sardinia data*): i-o summary statistics of association (regression coefficients and standard errors) obtained in an our previous study in which we analyzed Immunochip genotyping data from 15 multiplex Sardinian families (75 affected and 254 unaffected members), plus 94 unrelated healthy subjects from the same population using generalized estimating equation to allow for non-independence between members in the same family (Fazia et al., 2017).
(ii) *Dataset 2* (*UKBEC transcriptome data*): Immunochip genotyping and gene expression measurements made in a collection of autoptic tissue from ten brain regions (i.e., CRBL, FCTX, HIPP, MEDU, OCTX, PUTM, SNIG, THAL, TCTX and WHMT) of a sample of 134 confirmed control individuals free of any neuropathological disease of European descent. These data were provided by the UKBEC http://www.braineac.org (Trabzuni et al., 2011; Ramasamy et al., 2014). We obtained from these data the required i-e association statistics (regression coefficients and standard errors).

### Gene/Instruments Selection

We applied MR method to a restricted selected *a priori* set of genes (*CCL2*, *NFKB1*, *MAPK14*, *TNFRSF1A*, *CXCL10*) identified as plausible MS risk genes on the basis of previous, independent studies and of their involvement on NF-κB signaling pathway.

#### CCL2

C-C chemokine ligand 2 (CCL2 or MCP-1) is a pro-inflammatory chemokine that, through an interaction with its receptor CCR2, attracts dendritic cells, monocytes, T cells, and natural killer cells at inflammatory sites (Sorensen et al., 2004; Deshmane et al., 2009). *CCL2* expression is largely dependent on NF-κB signaling pathway (Hayden and Ghosh, 2012; Nakatsumi et al., 2017; Wang et al., 2018). *CCL2* mRNA was found significantly increased in demyelinated MS hippocampus with discrepancies in the spatial and quantitative distribution with respect to gray matter and white matter lesions (Prins et al., 2014). Furthermore, CCL2-induced monocyte migration results in blood brain barrier breakdown through the downregulation of endothelial tight junction proteins (dos Santos et al., 2005).

#### CXCL10

CXCL10 or IP-10 is a member of the CXC subtype of the chemokine superfamily and is expressed in astrocytes, glial cells, endothelial cells, macrophages, T cells, neutrophils, dendritic cells, keratinocytes, fibroblasts and hepatocytes (Kasama et al., 2011; Vazirinejad et al., 2014; Iwanowski et al., 2017). Its role in the pathogenesis of MS is based on chemoattracting Th1 to CNS (Dufour et al., 2002; Sørensen et al., 2002; Iwanowski et al., 2017). NF-κB transcriptional activation in endothelial and in microglia cells enhances *CXCL10* induction in response to tumor necrosis factor α (TNFα) (Harris et al., 2014). *CXCL10* expression takes place via the p38/MAPK, JNK/MAPK and NF-κB cascade (Shen et al., 2006; Harris et al., 2014; Jin et al., 2017).

#### MAPK14

*MAPK14* (p38α) belongs to mitogen-activated protein kinase (MAPK) family. Signaling via the MAPK14 pathway is important in the regulation of inflammatory response in multiple cell type and the production of specific cytokines and chemokines (Lo et al., 2015). Under CNS inflammatory condition, p38α-deficient mice show a reduced reactivity of astrogliosis and impairment in the formation of astroglial barrier, thus revealing the importance of p38α signaling in maintaining the barrier of CNS microcirculation (Lo et al., 2015). p38a signaling in astrocytes critically regulates specific subsets of cytokines (TNFα, IL-6) and chemokines (CXCL1, CXCL2, CXCL10, CCL2, CCL4). The result is that without the astroglial barrier an elevated number of macrophages/microglia in the CNS contributes to an increase in the upregulation of chemokines and cytokines leading to an uncontrolled inflammation.

#### NFKB1

*NFKB1* encodes the p50 subunit of NF-κB transcriptional complex. Several studies have found genetic variants within or near this gene to be associated with MS (International Multiple Sclerosis Genetics, 2011; International Multiple Sclerosis Genetics Consortium, Beecham et al., 2013; Hussman et al., 2016). Two *in vivo* studies showed that *NFKB1*-deficient mice are significantly resistant to EAE (Hilliard et al., 1999; Leibowitz and Yan, 2016), while mice knockout for the inhibitor of p50, *I*κ*B*α, are characterized by the constitutive activation of NF-κB in microglia/macrophages during EAE, developing an exacerbated EAE disease course with enhanced inflammatory infiltration and demyelination in the CNS (Yue et al., 2018).

#### TNFRSF1A

This gene encodes (TNF) receptor–1, an important player in MS susceptibility (Tienari and Hohlfeld, 2013). TNFRSF1A leads to the activation of different signaling pathways, such as NF–κB or MAPK pathways, two important pathways associated with MS susceptibility (Housley et al., 2015). GWAS have identified MS-associated variants within or proximal to TNFRSF1A (De Jager et al., 2009; International Multiple Sclerosis Genetics, 2011; International Multiple Sclerosis Genetics Consortium, Beecham et al., 2013; Andlauer et al., 2016; Hussman et al., 2016).

The MR approach requires that each of the five selected genes fulfill two important conditions:

(i) to have independent SNPs (r^2^ < 0.20) *in cis* and *in trans* throughout the genome associated with their levels of expression (analyzing UKBEC transcriptome data),
(ii) to have the same exposure-significant SNPs also genotyped in the Sardinia data and for which we have summary-level data of their association with MS.

We selected as IVs those variants whose regression coefficients of association with the gene expression level achieves a *p*-value < 5 × 10^–4^. The transcript-level expression profile used is estimated as the Windsorized mean of the exon-level probesets. The association test between each SNP and the transcript-level expression profile was performed for each of the ten available brain regions, by modeling the effect of genotype as additive linear. Because gene expression may be highly specific for a particular area of the brain, the importance of sampling different areas of the brain cannot be understated. In fact, in the analysis of each selected gene, we separately assessed the possible causal link between MS and changes in expression in each available different area of the brain. In each tissue, gene expression values were natural-log transformed and then standardized to have a more logical and useful interpretation of the causal effect, which represents the increase of the log-odds ratio due to an increase equal to 1 standard deviation (SD) of the log transformed gene expression.

### Univariable Mendelian Randomization

We estimated the total causal effect, that is composed by the direct and indirect effects, i.e., mediated by the others, of each gene in each brain region, using three MR methods: the Inverse-Variance Weighted estimator (IVW), the MR-Egger Regression estimator (MR-ER) and the Weighted Median estimator (WME). We use IVW for main analysis and MR-ER and WME for sensitivity analysis.

Here below we discuss IVW in detail being the main analysis and leave the reader to look at the cited literature for MR-ER and WME (Egger et al., 1997; Han, 2008; Bowden et al., 2016).

In brief, each j-th instrument, or variant Z_j_, with *j* = 1, … *J*, contributes a separate IV estimate of the causal effect of X on Y, given by the ratio ${\hat{\beta }}_{Y\,Z_{j}}/{\hat{\beta }}_{X\,Z_{j}}$, where ${\hat{\beta }}_{Y\,Z_{j}}$ is the estimated coefficient of Z_j_ in a univariate linear regression of Y on that instrument (log-odds ratio if Y is binary) and ${\hat{\beta }}_{X\,Z_{j}}$ is the estimated coefficient of X on the same instrument Z_j_ (log-odds ratio if X is binary). The IVW estimator of the causal effect of X on Y combines the IV estimates from multiple independent instruments that are assumed to satisfy all the IV conditions we described in the introduction, via the formula:

$${\hat{\beta }}_{I\,V\,W}=\frac{\Sigma_{j}\,{\hat{\beta }}_{X\,Z\,j}\,\sigma_{Y\,j}^{-2}\,{\hat{\beta }}_{Y\,Z\,j}}{\Sigma_{j}\,{\hat{\beta }}_{X\,Z\,j}^{2}\,\sigma_{Y\,j}^{-2}}$$

where σ*_*Yj*_* is the standard error of the gene-exposure association estimate for instrument *j* (Burgess et al., 2013; Bowden et al., 2016). If all genetics variants satisfy the IV assumptions, then the IVW estimate is a consistent estimate of the causal effect (e.g., it converges to the true value as the sample size increases), as it is a weighted mean of the individual ratio estimates. However, in the IVW method if only one genetic variant is not a valid IV, then the estimator is typically biased. Hence, two methods were used as a sensitivity analysis: MR-ER and WME. MR-ER allows all genetic variants to violate pleiotropy assumption, and the intercept from the MR-ER analysis can be interpreted as the average pleiotropic effect of genetic variants included in the analysis and a measure of potential bias affecting IVW estimate (Egger et al., 1997). However, this method requires all genetic variants to satisfy an alternative assumption (InSIDE assumption), valid only if pleiotropic effects are independently distributed from i-e association ${\hat{\beta }}_{X\,Z_{j}}$. In contrast, WME can be obtained using inverse-variance weights in a weighted median function; it relaxes MR assumptions and gives consistent estimates assuming that IVs representing over 50% of the weight are non-pleiotropic (Han, 2008; Bowden et al., 2016). The assumptions underlying each method are summarized in Table 1.

**TABLE 1:** Assumptions required by employed methods.

Method	Assumptions
IVW	All instruments must be mutually independent, non-pleiotropic, and satisfy conditions (*i*-*ii*)
WME	All instruments must be mutually independent and satisfy conditions (*i*-*ii*). The no-pleiotropy condition must be satisfied by a subset of instruments that accounts for at least 50% of the total weight (minor weight condition).
MR-ER	All instruments must be mutually independent and satisfy conditions (*i*-*ii*). InSIDE must hold.

*IVW, Inverse Variance Weighted; MR-ER, MR-Egger Regression; WME, Weighted Median Estimator.*

To assess the validity of the IVs, Cochrane’s Q test (heterogeneity test) was used to check the presence of heterogeneity, that indicates a possible violation of the IV assumptions (Greco et al., 2015). If the Cochrane’s Q statistics is large (yielding correspondingly small *p*-values), the estimated causal effects of the exposure may vary across the population or between variants. To identify IVs which give the largest contribution to Cochran’s Q statistic, IVW Radial-MR was performed. IVW Radial-MR weights causal estimates with the square root of the actual weight each SNP receives in the IVW analysis (Bowden et al., 2018). Q statistic obtained for each IV represents the contribution Q_*j*_ to the overall Q statistic. If the contribution was statistically significant at α = 0.05 then the IV was considered as highly potential invalid and thus removed, and the analysis repeated without the outlier to obtain a more reliable result.

To identify potentially invalid IVs, forest plots and scatter plots were used as visual tools: the forest plot shows the causal estimates (i.e., IVW and WME) expressed as log-odds ratio for each IVs with their 95% Confidence Interval (CI). The scatter plot shows ${\hat{\beta }}_{X\,G_{j}}$ and ${\hat{\beta }}_{Y\,G_{j}}$ respectively on X-axis and Y-axis, with θ_*IVW*_, θ_*WME*_ and θ_*MR–ER*_ as slopes allowing to evaluate MR-Egger intercept.

For all the analyses, we have reported causal estimates for IVW, MR-ER, and WME. The effect size was calculated as the effect of a 1 SD change in natural-log-transformed gene expression level, since this metric is more interpretable than an arbitrary difference.

### Multivariable Mendelian Randomization

In polygenic MR investigations, as ours, in which both *cis*- and *trans*-acting IVs are involved, while a high number of instruments results in an higher statistical power, the chance of including a pleiotropic variant is higher than using *cis*-variants from a single region (Brion et al., 2013). Pleiotropy is commonly classified as *vertical* and *horizontal*. In the *vertical pleiotropy*, genetic variants are associated with multiple exposures belonging to the same causal pathways, while in the *horizontal pleiotropy*, genetic variants are associated with multiple exposures belonging to different causal pathways. As discussed elsewhere pleiotropy may lead to biased estimate of causal effects (Bowden et al., 2015; Burgess and Thompson, 2015).

Given our studied genes belong to the same pathway they might have few instruments affecting more than one exposure and the use of MVMR allows us to control for *vertical pleiotropy.*

Furthermore, in our application we are also interested in estimating, in addition to the total effect, the direct effect of each specific gene expression. This is motivated by their likely relatedness due to being in the same pathway and exhibiting correlation as shown in Supplementary Figure S1 and Supplementary Table S1. As a consequence, many of them may exert a causal effect on the outcome, one mediating the effect of the other(s). MVMR by jointly estimating the causal effects of all exposures on the outcome allows: (i.) to estimate the direct effect of an exposure (within a mediation scenario), (ii.) to mitigate a possible *vertical pleiotropy* bias, due to instruments affecting more than one exposure.

The assumptions underlying MVMR are quite similar to those of standard MR i.e., each variant: (i) is associated with one or more exposures, (ii) is not associated with any confounders, and (iii) affects the outcome only via one or more of the exposures included in the model (Burgess and Thompson, 2015; Burgess et al., 2019). MVMR requires as instruments a composite set of SNPs, *Z*, associated with the exposures included in the model and that do not affect the outcome other than through these exposures. In our MVMR analysis we used as instruments the genetic instruments specific and common (composite) to the investigated exposures.

Within the MVMR scenario using summary-level data, IVW can be generalized by fitting the model with the intercept set to zero:

$${\hat{\beta }}_{Y\,Z_{j}}=\theta_{1}\,{\hat{\beta }}_{X_{1,Z\,j}}+\theta_{2}\,{\hat{\beta }}_{X_{2,Z\,j}}+\theta_{3}\,{\hat{\beta }}_{X_{3,Z\,j}}+\theta_{4}\,{\hat{\beta }}_{X_{4,Z\,j}}+\theta_{5}\,{\hat{\beta }}_{X_{5,Z\,j}}+\varepsilon_{j}$$

Where ${\hat{\beta }}_{Y\,Z_{j}}$ is the summary-level data of the association between each *j* genetic variant and the outcome, ${\hat{\beta }}_{1,Z_{j}}$ is the genetic effect of Zj on X_1_; while θ_*1*_ is the direct causal effect of interest for X_1_. The MVMR Egger (MVMR-ER) uses the same regression model but allowing the intercept to be estimated. To assess instrument validity we used an adjusted version of the Cochran Q statistic (Q_A_) proposed by Sanderson (Sanderson et al., 2019). Excessive heterogeneity in Q_*A*_ brings assumptions *ii*) and *iii.*) (detailed in methods section) into doubt. To identify specific genetic variants as outliers we used each variant’s contribution *q*, defined as the weighted squared difference between the observed and predicted association with the outcome, to the overall Q_A_ (Zuber et al., 2020). If the *q* contribution was statistically significant different from zero (*p*-value < 0.05) then the genetic variant was considered as highly potential invalid instrument and thus removed, and the analysis conducted without that variant to obtain a more reliable result.

All the analyses were performed using TwoSampleMR (Yavorska and Burgess, 2017), RadialMR (Bowden et al., 2018) and MendelianRandomization (Burgess et al., 2015) R packages. Benjamini-Hochberg correction (Yekutieli and Benjamini, 2001), fixing the False Discovery Rate (FDR) at α < 0.05 was used to account for multiple comparison. To further confirm our results, we used a bootstrap procedure for both univariable and multivariable analysis. Since we are using summary-data, to obtain MR univariable bootstrap confidence intervals we generated 1000 datasets, in which each i-e association ${\dot{\beta }}_{X,Z_{j}}$ has been sampled from a normal distribution with mean equal to ${\hat{\beta }}_{X,Z_{j}}$ and standard deviation equal to se$\left({\hat{\beta }}_{\text{X},\text{Zj}}\right)$. In the multivariable scenario we generated 1000 datasets, in which each i-e association ${\dot{\beta }}_{X_{k},Z_{j}}$, for each of the *k* = 5 genes, has been sampled from a normal multivariable distribution with means equal to the vector of the 5 i-e associations ${\hat{\beta }}_{X_{k},Z_{j}}$ and covariance matrix equal to the covariance matrix between gene expressions in that specific tissue. MR (and MVMR in the multivariable scenario) analysis was conducted for each of the 1000 datasets generated, so that 1000 IVW estimates were obtained. Using the percentile method, the confidence interval is given by 95% central in the distribution of bootstrap replications. That is, the plausible IVW estimates are those falling between the 2.5-th percentile and the 97.5-th percentile of the bootstrap distribution estimates. We considered as statistically significant those results reaching FDR adjusted *p*-value < 0.05 and whose bootstrap confidence intervals does not contain the estimate under the null.

**FIGURE 1:**
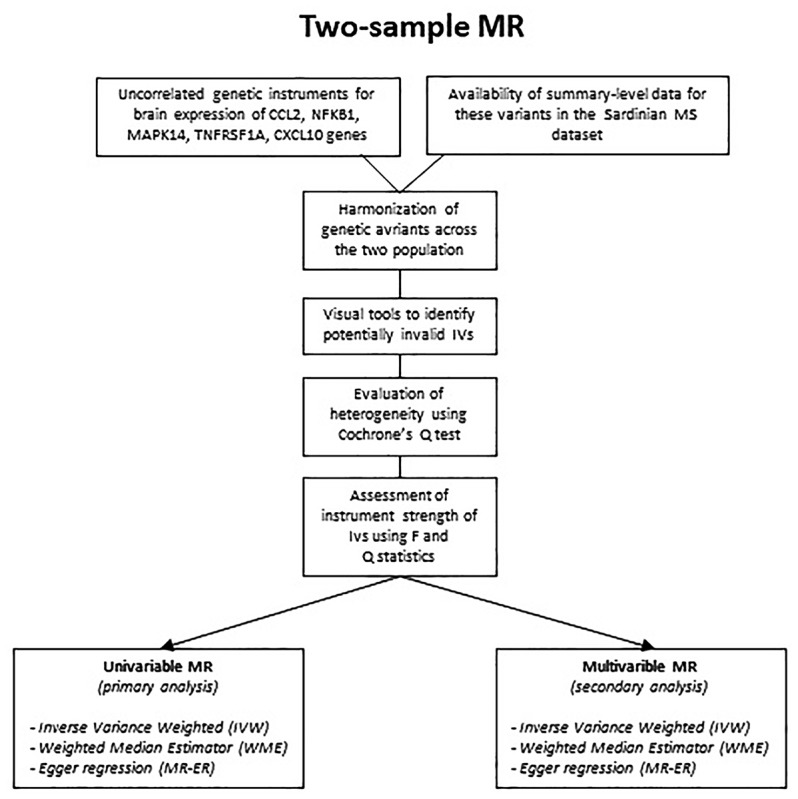
Flowchart of the analysis.

**FIGURE 2:**
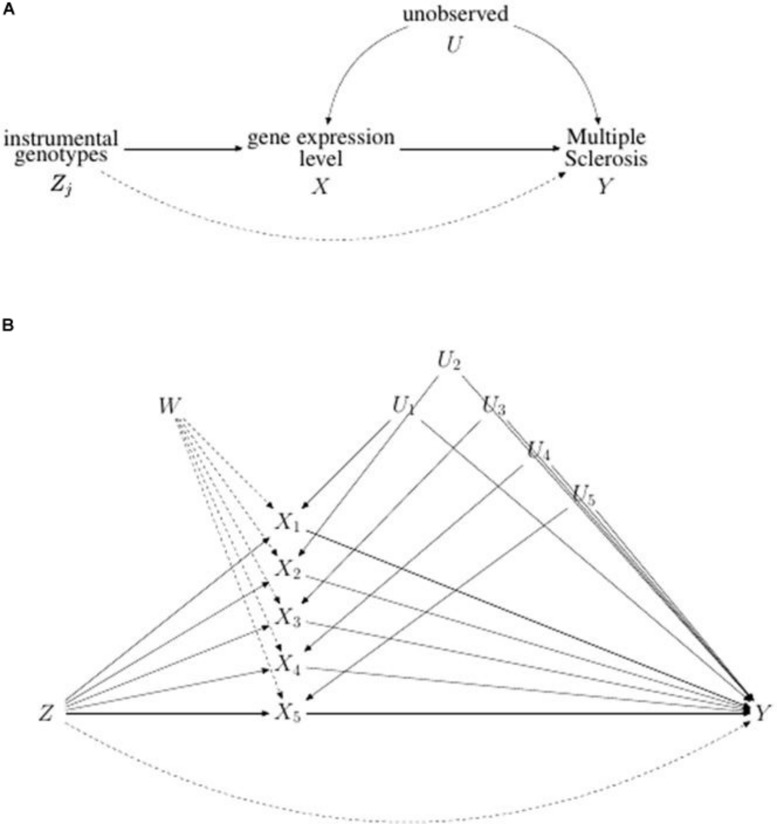
Directed acyclic graph illustrating **(A)** the univariable and **(B)** the multivariable Mendelian Randomization problem. In **(A)** the potential violation of IV assumption represented by the direct pleiotropic effect of *Zj* on *Y* is indicated by a dotted line. In **(B)** causal graph illustrating multivariable Mendelian randomization assumptions for a set of genetic variants *Z*, five risk factors *X*_1_, *X*_2_, *X*_3_, *X*_4_ and *X*_5_, and the binary outcome Y. The direct pleiotropic effect of *Z* on *Y* is represented by a dotted line. The node *W* represents the possibility that each risk factor, *X*, could influence each other.

Flowchart of the analysis is reported in Figure 1, while causal diagram for (A) univariable MR and (B) MVMR scenario are reported Figure 2.

## Results

### Univariable MR

We analyzed five *a priori* selected genes (i.e., *CCL2*, *NFKB1*, *MAPK14*, *TNFRSF1A*, *CXCL10*) in ten different brain regions (i.e., CRBL, FCTX, HIPP, MEDU, OCTX, PUTM, SNIG, THAL, TCTX and WHMT). Table 2 reports for each gene in each brain tissue the number of IVs, the IVW, WME and MR-ER causal effect estimates, their corresponding standard error and *p*-value. Table 2 also reports in the last column the IVW estimate and its 95% confidence intervals obtained via bootstrap. In Supplementary Table S2 detailed information for the selected IVs in each brain tissue (e.g., summary statistics of the association between the instrument and the disease and between the instrument and gene expression, Q statistics, and f statistics) are reported.

**TABLE 2:** Result of Univariable MR analysis.

Gene	Tissue	N. of IVs	IVW- β	IVW- se	IVW- *p*-value	WME- β	WME- se	WME- *p*-value	MR-ER - β	MR-ER- se	MR-ER- *p*-value	IVW Bootstrap - β [CI]
*CCL2*	FCTX	13	0.064	00.084	0.444	–0.013	0.120	0.914	0.407	0.297	0.198	0.058[−0.102;0.234]
	CRBL	9	–0.034	0.106	0.747	–0.049	0.140	0.727	0.243	0.440	0.599	−0.037[−0.246;0.161]
	OCTX	10	0.257	0.096	0.007	0.224	0.131	0.087	0.552	0.496	0.297	0.246[0.058;0.443]
	PUTM	10	0.111	0.098	0.255	0.210	0.130	0.107	0.245	0.384	0.542	0.107[−0.076;0.291]
	WHMT	6	–0.044	0.146	0.761	–0.141	0.188	0.453	–0.122	0.708	0.871	−0.041[−0.326;0.255]
	SNIG	13	0.076	0.079	0.336	0.137	0.107	0.201	0.435	0.321	0.203	0.072[−0.074;0.226]
	THAL	10	0.197	0.112	0.079	0.377	0.146	0.010	0.143	0.641	0.829	0.191[−0.011;0.397]
	HIPP	4	–0.028	0.196	0.887	–0.006	0.240	0.981	0.704	0.796	0.470	−0.181[−0.542;0.151]
	MEDU	11	0.272	0.083	0.001	0.276	0.133	0.039	0.217	0.267	0.436	0.260[0.092;0.437]
	TCTX	11	–0.008	0.091	0.932	0.127	0.127	0.318	–0.097	0.443	0.832	−0.007[−0.191;0.184]

*CXCL10*	FCTX	7	0.079	0.141	0.574	0.194	0.185	0.294	0.835	0.565	0.200	0.077[−0.214;0.365]
	CRBL	10	0.173	0.100	0.084	0.261	0.136	0.056	0.291	0.317	0.386	0.166[−0.019;0.369]
	OCTX	13	–0.062	0.095	0.514	–0.179	0.136	0.189	–0.745	0.350	0.057	−0.063[−0.249;0.127]
	PUTM	13	–0.161	0.089	0.070	–0.134	0.126	0.287	0.111	0.440	0.806	−0.150[−0.324;0.031]
	WHMT	12	0.066	0.086	0.445	0.073	0.109	0.504	0.135	0.373	0.725	0.060[−0.109;0.233]
	SNIG	7	–0.060	0.083	0.468	–0.051	0.109	0.636	–0.301	0.265	0.307	−0.064[−0.227;0.096]
	THAL	19	0.128	0.066	0.054	0.016	0.102	0.875	0.111	0.237	0.646	0.122[−0.006;0.255]
	HIPP	14	0.037	0.101	0.713	0.202	0.129	0.117	–0.175	0.395	0.665	0.032[−0.147;0.212]
	MEDU	7	0.080	0.095	0.400	–0.036	0.132	0.784	–0.318	0.462	0.522	0.075[−0.103;0.273]
	TCTX	17	0.001	0.073	0.991	–0.041	0.099	0.677	–0.232	0.240	0.348	−0.002[−0.136;0.131]

*MAPK14*	FCTX	12	–0.135	0.085	0.111	–0.249	0.120	0.038	–0.350	0.294	0.262	−0.127[−0.286;0.041]
	CRBL	7	0.010	0.126	0.938	–0.019	0.167	0.909	–0.354	0.550	0.548	0.007[−0.235;0.258]
	OCTX	16	–0.165	0.096	0.085	–0.243	0.136	0.073	–0.261	0.354	0.473	−0.154[−0.336;0.031]
	PUTM	12	–0.159	0.085	0.060	–0.179	0.116	0.124	–1.641	0.837	0.078	−0.49[−0.315;0.016]
	WHMT	6	–0.400	0.187	0.033	–0.182	0.225	0.419	0.528	0.490	0.341	−0.379[−0.731;−0.067]
	SNIG	18	–0.023	0.067	0.725	–0.050	0.097	0.608	0.407	0.289	0.179	−0.023[−0.160;0.116]
	THAL	9	–0.083	0.139	0.551	–0.113	0.180	0.529	–0.212	0.461	0.659	−0.079[−0.346;0.170]
	HIPP	14	–0.098	0.076	0.198	–0.024	0.101	0.809	–0.191	0.454	0.682	−0.097[−0.258;0.059]
	MEDU	8	–0.083	0.098	0.397	–0.114	0.136	0.402	0.194	0.329	0.577	−0.083[−0.286;0.114]
	TCTX	13	–0.113	0.090	0.208	–0.111	0.122	0.364	0.071	0.342	0.838	−0.108[−0.276;0.057]

*NFKB1*	FCTX	9	0.306	0.127	0.016	0.253	0.177	0.152	0.706	0.563	0.250	0.290[0.042;0.545]
	CRBL	9	0.332	0.103	0.001	0.421	0.144	0.003	0.452	0.360	0.250	0.312[0.103;0.543]
	OCTX	15	–0.025	0.072	0.724	0.009	0.100	0.926	–0.193	0.265	0.479	−0.025[−0.156;0.102]
	PUTM	16	–0.069	0.082	0.405	–0.115	0.115	0.317	0.716	0.529	0.198	−0.065[−0.219;0.107]
	WHMT	13	–0.037	0.091	0.684	–0.029	0.128	0.821	–0.676	0.548	0.243	−0.037[−0.222;0.142]
	SNIG	14	–0.090	0.080	0.259	–0.048	0.111	0.667	0.469	0.420	0.286	−0.081[−0.238;0.063]
	THAL	11	0.134	0.101	0.186	0.127	0.129	0.325	0.321	0.458	0.501	0.126[−0.077;0.347]
	HIPP	11	–0.121	0.096	0.207	–0.092	0.136	0.500	0.262	0.465	0.587	−0.118[−0.317;0.061]
	MEDU	12	0.023	0.099	0.815	–0.045	0.137	0.744	0.127	0.485	0.799	0.021[−0.172;0.221]
	TCTX	16	–0.112	0.083	0.178	–0.104	0.116	0.373	0.158	0.375	0.681	−0.105[−0.262;0.062]

*TNFRS1A*	FCTX	17	–0.038	0.075	0.611	0.030	0.108	0.781	–0.342	0.310	0.287	−0.037[−0.187;0.101]
	CRBL	14	0.037	0.090	0.683	0.038	0.125	0.761	–0.158	0.298	0.604	0.037[−0.137;0.213]
	OCTX	11	0.097	0.095	0.312	0.101	0.129	0.434	0.205	0.275	0.474	0.093[−0.094;0.277]
	PUTM	11	0.096	0.082	0.237	0.085	0.111	0.443	–0.231	0.379	0.557	0.092[−0.065;0.248]
	WHMT	12	0.202	0.101	0.046	0.212	0.135	0.116	0.062	0.401	0.880	0.191[−0.0001;0.340]
	SNIG	8	–0.173	0.109	0.111	–0.177	0.147	0.231	0.376	1.190	0.763	−0.165[−0.388;0.051]
	THAL	16	0.153	0.085	0.072	0.030	0.121	0.803	–0.113	0.341	0.744	0.147[−0.028;0.324]
	HIPP	11	0.051	0.101	0.614	0.112	0.131	0.392	–0.601	0.456	0.221	0.043[−0.159;0.246]
	MEDU	11	0.150	0.097	0.121	0.259	0.123	0.035	0.259	0.295	0.402	0.142[−0.028;0.319]
	TCTX	10	–0.321	0.113	0.005	–0.359	0.157	0.022	–0.085	0.574	0.886	−0.312[−0.550;−0.102]

*The table reports for each gene and for each tissue, the number of IVs, the causal effect β, its standard error and p-value for IVW, WME and MR-ER methods. In the last column the IVW estimate and its 95% confidence intervals obtained via bootstrap are also reported. CRBL, cerebellum; FCTX, frontal cortex; HIPP, hippocampus; MEDU, medulla; OCTX, occipital cortex; PUTM, putamen; SNIG, substantia nigra; THAL, thalamus; TCTX, temporal cortex; WHMT, intralobular white matter. IVW, Inverse Variance Weighted; WME, Weighted Median Estimator; MR-ER, Egger estimator.*

An estimate was considered as statistically significant, if the FDR corrected *p*-value was < 0.05, and its 95% bootstrap confidence interval did not include the value of the estimate under the null. According to this criterion *CCL2* gene in MEDU and *NFKB1* gene in CRBL turned out statically significant. These results are discussed in detail in the following part of the text.

#### CCL2 Expression in Medulla

Mendelian Randomization analysis has been conducted using 11 IVs. IVW estimate ${\hat{\theta }}_{I\,V\,W}$ was 0.272 (C.I. 95% [0.109, 0.435]) with exp $\left({\hat{\theta }}_{I\,V\,W}\right)$ leading to an OR = 1.31 (95% CI [1.12; 1.54]). This suggests that an increase of natural log-transformed *CCL2* expression by 1 SD (0.15) in MEDU causes an increase in the odds to develop MS. Sensitivity analysis with WME led to an almost identical estimate (${\hat{\theta }}_{W\,M\,E}$ = 0.276) that resulted statistically significant different from zero (*p*-value < 0.039). MR-ER does not give enough evidence of a directional pleiotropy since the intercept estimate was not statistically significant being its value close to zero (α = −0.046, *p*-value > 0.05); while causal effect estimate was slightly lower, but still similar in magnitude (${\hat{\theta }}_{M\,R-E\,R}$ = 0.217). Cochrane’s Q test did not give evidence of heterogeneity between the instruments (*Q* = 7.90, df = 10, *p*-value = 0.64). Radial-MR did not show any instrument to statistically significant contribute to heterogeneity. Thus, these results are robust to the sensitivity analysis and hence give a hint of a potential total causal effect on MS of *CCL2* expression in MEDU.

Bootstrap IVW estimate was equal to 0.260 with 95% CI [0.092;0.437], thus giving the same evidence.

**FIGURE 3:**
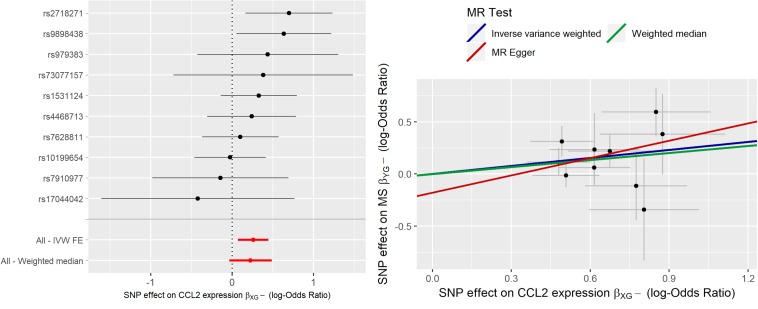
Forest plot and Scatter plot of causal estimates of *CCL2* expression in medulla (MEDU) on MS.

In Figure 3 the forest plot and the scatter plot of causal estimates of *CCL2* expression in MEDU on MS are shown.

#### *NFKB1* Expression in Cerebellum

**FIGURE 4:**
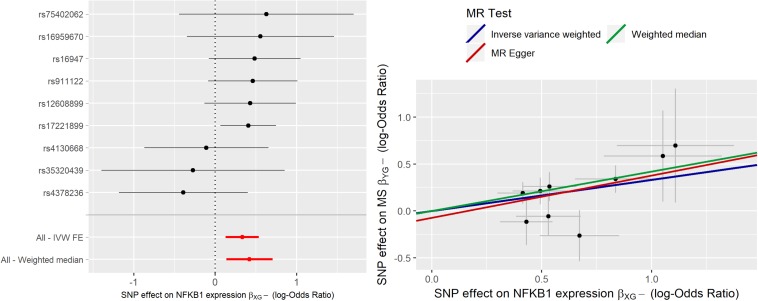
Forest plot and Scatter plot of causal estimates of *NFKB1* expression in cerebellum (CRBL) on MS.

MR analysis was initially carried out using 10 IVs. IVW estimate of *NFKB1* causal effect was ${\hat{\theta }}_{I\,V\,W}$ = 0.281 (95% CI [0.085;0.477]); sensitivity analysis with WME led to a higher estimate (${\hat{\theta }}_{W\,M\,E}$ = 0.417), while MR-ER gave a similar estimate, (${\hat{\theta }}_{M\,R-E\,R}$ = 0.237) with no evidence of a directional pleiotropy [intercept estimate was not statistically significant (α = −0.027, *p*-value > 0.05)]. Radial-MR identified an instrument contributing to heterogeneity (i.e., rs6109595 with *Q* = 3.98, *p*-value = 0.046). This instrument appears to be potentially invalid, as also confirmed by the forest plot and the scatter plot, where the causal estimate of this SNP appears to be far from the others, and to “move” the IVW estimate toward the null. Thus, we considered this instrument as pleiotropic and, given the statistical evidence by Radial-MR, and we removed it from the analysis. In Figure 4 the forest plot and the scatter plot of causal estimates of *NFKB1* expression in CRBL on MS with the presence of the potentially invalid IV are reported.

After the outlier’s removal, IVW estimate ${\hat{\theta }}_{I\,V\,W}$ = 0.332 (95% CI [0.129;0.534]) and WME estimate (${\hat{\theta }}_{W\,M\,E}$ = 0.421) turned out more similar in magnitude. WME estimated obtained by including the outlier IV was very similar to that obtained by excluding it; this can be explained by the fact that WME relaxes (*ii*) and (*iii*) assumptions so that IVs representing more than 50% of the weight need to be valid IVs. Since the outlier SNP did not weight more than 50%, its removal did not affect the causal estimate. After outlier’s removal, the MR-ER estimate (${\hat{\theta }}_{M\,R-E\,R}$ = 0.452) turned out being very close to the WME estimate, thus indicating potential residual bias in IVW estimate due to presence of pleiotropic SNPs. The scatter plot clearly shows how the slopes of the WME and MR-ER are almost perfectly overlapping while IVW slope is slightly lower. Moreover, there is no evidence of directional pleiotropy (MR-ER intercept close to 0, α = −0.073, *p*-value > 0.05) or heterogeneity (*Q* statistic = 6.93, *p*-value = 0.54). Exp $\left({\hat{\theta }}_{I\,V\,W}\right)$ corresponding to an OR = 1.39 (95% [1.14;1.71]), suggests that an increase of natural log transformed *NFKB1* expression by 1 SD (0.04) in CRBL causes an increased odds to develop MS. We conclude that these results show evidence of a potential total causal effect of *NFKB1* expression in CRBL on MS, since all the estimators give very similar causal effect estimates.

Bootstrap IVW estimate was also equal to 0.312 with 95% CI [0.103;0.543].

**FIGURE 5:**
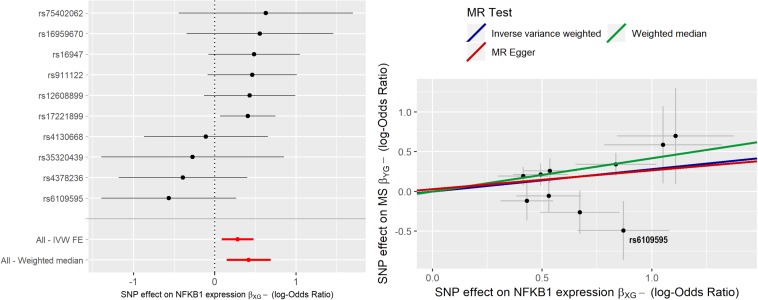
Forest plot and Scatter plot of causal estimates of *NFKB1* expression in cerebellum (CRBL) on MS after outlier’s removal.

Figure 5 reports the forest plot and scatter plot of the causal estimates of *NFKB1* expression in CRBL on MS after removing the outlier.

Table 3 reports the significant results for *CCL2* and *NFKB1*, which turned out as statistically significant after multiple testing correction.

**TABLE 3:** Statistically significant total causal effect for *CCL2* in MEDU and *NFKB1* in CRBL after FDR multiple testing correction.

Gene	Tissue	OR	95% confidence interval	Gene expression mean (on log scale)	Gene expression SD (0n log scale)
*CCL2*	MEDU	1.31	1.12, 1.54	1.84	0.15
*NFKB1*	CRBL	1.39	1.14, 1.71	1.81	0.04

*The table reports for each gene, the causal effect expressed as OR, its 95% CI, the mean gene expression and its SD on a log scale in the healthy individuals. Odds Ratios (OR) and 95% Confidence Intervals of causal estimates with mean and standard deviation of natural-log gene expression.*

### Multivariable MR

The fives selected genes (i.e., *CCL2*, *NFKB1*, *MAPK14*, *TNFRSF1A*, *CXCL10*) were subsequently analyzed jointly in a MVMR framework. Table 4 reports for each gene in each brain tissue the number of IVs, the MVMR-IVW and MVMR-ER direct causal effect estimates, their corresponding standard errors and unadjusted *p*-values; the last columns report the MVMR-IVW causal estimate and 95% confidence intervals obtained via bootstrap.

**TABLE 4:** Direct effect of the 5 genes in each brain region via Multivariable MR analysis.

Tissue	N. of IVs	Gene	IVW- β	IVW- se	IVW- *p*-value	MVMR-ER β	MVMR-ER- se	MVMR-ER- *p*-value	IVW Bootstrap - β [CI]
FCTX	58	*TNFRSF1A*	–0.089	0.150	0.551	–0.089	0.150	0.551	−0.084[−0.325;0.157]
		*MAPK14*	–0.129	0.207	0.533	–0.129	0.207	0.533	−0.157[−0.496;0.152]
		*CXCL10*	–0.057	0.143	0.690	–0.057	0.143	0.690	0.023[−0.215;0.255]
		*NFKB1*	0.222	0.224	0.322	0.222	0.224	0.322	0.165[−0.186;0.516]
		*CCL2*	0.017	0.118	0.670	0.050	0.118	0.670	0.026[−0.162;0.216]

CRBL	49	*TNFRSF1A*	0.366	0.148	0.013	0.421	0.156	0.007	0.357[0.096;0.619]
		*MAPK14*	–0.443	0.254	0.081	–0.479	0.256	0.061	−0.463[−0.879;−0.038]
		*CXCL10*	0.056	0.134	0.678	0.108	0.143	0.449	0.043[−0.181;0.309]
		*NFKB1*	0.749	0.258	0.004	0.716	0.260	0.006	0.762[0.355;1.174]
		*CCL2*	–0.521	0.172	0.002	–0.450	0.185	0.015	−0.517[−0.833;−0.239]

OCTX	64	*TNFRSF1A*	–0.049	0.128	0.705	–0.010	0.133	0.937	−0.020[−0.233;0.193]
		*MAPK14*	–0.173	0.175	0.321	–0.233	0.184	0.204	−0.081[−0.383;0.209]
		*CXCL10*	–0.080	0.091	0.380	–0.082	0.091	0.366	−0.026[−0.179;0.130]
		*NFKB1*	0.055	0.154	0.722	0.105	0.161	0.515	0.011[−0.269;0.304]
		*CCL2*	0.234	0.118	0.049	0.256	0.120	0.033	0.123[−0.100;0.340]

PUTM	61	*TNFRSF1A*	0.181	0.191	0.345	0.183	0.193	0.344	0.104[−0.222;0.411]
		*MAPK14*	0.064	0.228	0.777	0.034	0.268	0.898	0.024[−0.367;0.416]
		*CXCL10*	–0.023	0.118	0.848	–0.015	0.124	0.903	0.014[−0.169;0.216]
		*NFKB1*	–0.161	0.203	0.427	–0.132	0.245	0.592	−0.087[−0.440;0.241]
		*CCL2*	0.112	0.144	0.437	0.119	0.149	0.425	0.033[−0.195;0.272]

WHMT	46	*TNFRSF1A*	0.290	0.168	0.106	0.283	0.200	0.156	0.140[−0.202;0.474]
		*MAPK14*	0.029	0.219	0.899	0.026	0.235	0.912	0.005[−0.402;0.391]
		*CXCL10*	0.076	0.123	0.551	0.073	0.132	0.578	0.037[−0.207;0.259]
		*NFKB1*	–0.225	0.175	0.226	–0.226	0.188	0.230	−0.098[−0.422;0.255]
		*CCL2*	–0.207	0.198	0.317	–0.209	0.211	0.321	−0.096[−0.445;0.270]

SNIG	56	*TNFRSF1A*	–0.301	0.142	0.034	–0.107	0.168	0.526	−0.127[−0.383;0.114]
		*MAPK14*	–0.111	0.138	0.421	–0.089	0.139	0.523	−0.047[−0.276;0.187]
		*CXCL10*	–0.074	0.082	0.370	–0.028	0.086	0.740	−0.034[−0.176;0.113]
		*NFKB1*	0.084	0.154	0.586	0.159	0.158	0.314	0.035[−0.222;0.286]
		*CCL2*	0.273	0.118	0.021	0.271	0.118	0.022	0.114[−0.086;0.325]

THAL	62	*TNFRSF1A*	–0.038	0.173	0.827	–0.045	0.191	0.813	−0.011[−0.293;0.237]
		*MAPK14*	–0.359	0.193	0.062	–0.362	0.194	0.063	−0.187[−0.478;0.114]
		*CXCL10*	–0.024	0.113	0.830	–0.029	0.124	0.815	0.009[−0.179;0.192]
		*NFKB1*	0.356	0.166	0.032	0.357	0.166	0.032	0.181[−0.069;0.458]
		*CCL2*	0.082	0.197	0.678	0.081	0.198	0.685	0.053[−0.282;0.404]

HIPP	55	*TNFRSF1A*	–0.036	0.174	0.835	–0.016	0.198	0.934	−0.017[−0.286;0.249]
		*MAPK14*	–0.178	0.142	0.209	–0.192	0.156	0.219	−0.068[−0.286;0.147]
		*CXCL10*	–0.032	0.136	0.814	–0.025	0.141	0.861	−0.008[−0.199;0.176]
		*NFKB1*	0.158	0.162	0.329	0.179	0.188	0.343	0.049[−0.205;0.296]
		*CCL2*	–0.012	0.229	0.958	–0.015	0.232	0.947	0.006[−0.305;0.320]

MEDU	48	*TNFRSF1A*	–0.178	0.177	0.316	–0.270	0.214	0.208	−0.179[−0.479;0.153]
		*MAPK14*	0.142	0.201	0.478	0.158	0.203	0.436	0.127[−0.212;0.464]
		*CXCL10*	–0.093	0.122	0.445	–0.080	0.124	0.515	−0.090[−0.309;0.127]
		*NFKB1*	–0.021	0.211	0.921	–0.057	0.217	0.794	−0.012[−0.378;0.344]
		*CCL2*	0.404	0.189	0.032	0.436	0.194	0.025	0.396[0.045;0.736]

TCTX	58	*TNFRSF1A*	–0.190	0.210	0.366	–0.316	0.209	0.131	−0.065[−0.388;0.263]
		*MAPK14*	–0.031	0.170	0.857	0.186	0.176	0.291	0.002[−0.266;0.296]
		*CXCL10*	0.037	0.135	0.786	0.025	0.130	0.845	0.014[−0.204;0.230]
		*NFKB1*	–0.162	0.194	0.404	–0.289	0.195	0.138	−0.074[−0.388;0.256]
		*CCL2*	0.117	0.199	0.557	0.049	0.193	0.800	0.035[−0.333;0.369]

*The table reports for each gene and for each tissue, the number of IVs, the causal effect β, its standard error and *p*-value for IVW, and MR-ER methods. In the last column the IVW estimate and its 95% confidence intervals obtained via bootstrap are also reported. CRBL, cerebellum; FCTX, frontal cortex; HIPP, hippocampus; MEDU, medulla; OCTX, occipital cortex; PUTM, putamen; SNIG, substantia nigra; THAL, thalamus; TCTX, temporal cortex; WHMT, intralobular white matter; IVW, Inverse Variance Weighted; MVMR-ER, Egger estimator.*

Significant positive direct causal effects, also according to bootstrap estimates and 95% CIs, were obtained in CRBL for *NFKB1* (${\hat{\theta }}_{M\,V\,M\,R-I\,V\,W}$ = 0.749, *p*-value = 0.004, OR = 2.12, 95% CI [1.26;3.51]), *TNFRSF1A* (${\hat{\theta }}_{M\,V\,M\,R-I\,V\,W}$ = 0.366, *p*-value = 0.013, OR = 1.44, 95% CI [1.08;1.92]), while a negative direct causal effect was obtained for *CCL2* (${\hat{\theta }}_{M\,V\,M\,R-I\,V\,W}$ = −0.521, *p*-value = 0.002, OR = 0.59, 95% CI [0.42;0.83]), no direct causal effect was observed in CRBL for *MAPK14* and *CXCL10* genes.

A direct causal effect, also according to bootstrap estimates and CIs, was also observed in MEDU for *CCL2* gene (${\hat{\theta }}_{M\,V\,M\,R-I\,V\,W}$ = 0.404, *p*-value = 0.032, OR = 1.50, 95% CI [1.04;2.17]), no statistically significant direct causal effect was observed in MEDU for the remaining genes.

As regard MVMR-ER, there is no evidence of directional pleiotropy since the intercept is close to 0 and *p*-value is > 0.05. Heterogeneity tests do not show statistically significance, indicating that there is not variability in the genetic associations with the disease and likely there is no residual pleiotropy.

In Supplementary Table S3 detailed information for the selected IVs is reported.

Table 5 reports for *CCL2* in MEDU and for *NFKB1* in CRBL the gene expression mean level and the level of gene expression increased by 1 and 2 SD, that leads to an increased odds to develop MS. For CCL2 expression in MEDU the difference between total and direct effects is relatively moderate (31% vs 50%), while there is a big difference in NFKB1 expression (39% vs 111%). The direct effect of *CCL2* and *TNFRSF1A* in CRBL is also reported: an increase by 1 SD in CCL2 gene expression in CRBL seems to cause a decreased odds in developing the disease by 41%, while an increase in *TNFRSF1A* causes an increased odds of 44%. These values have been calculated on log-scale and then reverted back to the original scale.

**TABLE 5:** Increase in MS risk caused by increase of 1 and 2 SD of gene expression mean.

Effect	Gene	Tissue	Gene expression mean	Gene expression level after increase by 1 SD	Increase in risk	Gene expression level after increase by 2 SD	Increase in risk
Total	*CCL2*	MEDU	6.40	7.44	31%	8.64	62%
Direct	*CCL2*	MEDU	6.40	7.44	50%	8.64	100%
Total	*NFKB1*	CRBL	6.09	6.34	39%	6.60	78%
Direct	*NFKB1*	CRBL	6.09	6.34	111%	6.60	222%
Direct	*CCL2*	CRBL	4.50	5.00	−41%	5.56	−82%
Direct	*TNFRSF1A*	CRBL	5.42	5.72	44%	6.05	88%

## Discussion

The genetic architecture of MS susceptibility has been further characterized in the recently published genomic map of MS comprising 32 loci in the MHC and 200 non-MHC autosomal loci. This study has shown that these loci exert their effect in multiple different peripheral immune cells as well as in microglia, highlighting the importance of several cells of the peripheral and brain resident immune system. Thus the MS genetic risk is characterized as “*an autoimmune inflammatory process that targets CNS and triggers a neurodegenerative cascade*” (International Multiple Sclerosis Genetics Consortium, 2019).

Large GWASs have identified a wealth of SNPs responsible of genetic susceptibility to complex diseases, such as MS, and many of these signals could operate via gene expression regulation, given the majority of them are located in non-coding genomic regions (Barr and Misener, 2016).

In the last years understanding how associated genetic variations contribute to the biological pathways involved in the progression and pathogenesis of diseases has become an achievable goal for genetic research given the availability of public gene expression database in different tissues and genotyping database, together with the development of appropriate statistical methods. More specifically, causal inference framework plays an important role in this respect since it allows to answer questions such as “*does variation in the expression of a given gene influences the disease risk?*” and not simply “*the expression of a given gene influences the disease”*. The identification of a disease-associated SNP being at the same time an eQTL does not imply that the expression of the involved gene is causally related to the disease risk. In our strategy we applied MR approach to investigate the causal relationship between the expression of a given gene and MS.

In our work, in the effort to investigate and understand MS etiology, we focused on five genes related to the pro-inflammatory NF-κB signaling pathway (i.e., *CCL2*, *NFKB1*, *MAPK14*, *TNFRSF1A*, *CXCL10*), given the observation that this pathway is out of balance in MS and could represent a valid therapeutic target via inhibiting proper molecules implicated in it. NF-κB acts on many immune cells, and its constitutive activation leads to an increased inflammation in inflammatory and autoimmune diseases, such as MS (Yan and Greer, 2008). Furthermore, NF-κB emerged in the pathway enrichment for the prioritized genes of the 200 non-MHC loci in the last IMSGC paper (International Multiple Sclerosis Genetics Consortium, 2019).

We investigated the effect of each of these specific genes on MS susceptibility by performing a two-sample MR analysis, using summary-level information from two distinct datasets (i.e., Sardinia and UKBEC data). Sardinia data consists in the summary statistics of association between Immunochip SNPs and MS, while UKBEC data consists in transcript-level expression profile data on ten different brain tissues in addition to Immunochip data for 134 subjects free of neurological disorders. We performed both a univariable and a multivariable MR: the first approach was used to estimate the total causal effect of each exposure and the second to estimate the direct causal effect of each exposure by allowing multiple risk factors to be modeled at once, thus also allowing for measured pleiotropy.

In the univariable analysis two gene expressions showed significant results after multiple testing correction: *CCL2* in MEDU and *NFKB1* in CRBL tissue. These findings, also emerged as robust with sensitivity analysis and further confirmed using bootstrap procedure, are consistent with previous literature discussed below.

CCL2 is a chemokine involved in immunoregulatory and inflammatory processes, and its expression in astrocytes during EAE was found to be functionally significant, playing a role in the infiltration of macrophage and T cell in the CNS, and in the diffuse activation of astrocytes and microglia in both white and gray matter. Higher *CCL2* expression correlates also with relapses, more severe EAE clinical scores, demyelination and axonal loss (Kim et al., 2014). A less severe disease course was observed after induction of EAE in mice with a conditional, astrocyte-specific gene deletion of *CCL2* (Ponath et al., 2018b), while endothelial *CCL2* knockout mice were observed to be resistant to EAE (Ge et al., 2009). Our analysis shows that an increased expression of *CCL2* in MEDU is causally related with MS (OR = 1.31; 95% CI [1.12;1.54]), consistent with the above described results, and also ascribing a causative role to *CCL2* expression, rather than a simple association with MS.

*NFKB1* gene encodes p50 subunit of NF-κB transcriptional protein complex, an important complex involved in immune responses. *NFKB1* variants were found to be associated in GWASs with MS, and, in particular, two variants were found to increase gene expression (Housley et al., 2015; Ponath et al., 2018a). Furthermore, *NFKB1*-deficient mice are significantly resistant to EAE (Hilliard et al., 1999; Leibowitz and Yan, 2016), and mice knockout for the inhibitor of p50, *I*κ*B*α, are characterized by the constitutive activation of NF-κB in microglia/macrophages during EAE, developing an exacerbated EAE disease course with enhanced inflammatory infiltration and demyelination in the CNS (Yue et al., 2018). Our results are consistent with these biological evidences and attribute a causative role of increased *NFKB1* expression in CRBL on MS (OR = 1.39; 95% CI [1.14;1.71]).

A possible explanation of both *CCL2* and *NFKB1* expression causative role could be an abnormal response of astrocytes to myelin damage. More specifically, NF-κB pathway is an active pathway in astrocytes during myelin repair (with CCL2 as one of the products of this pathway). Furthermore, during myelin debris damage, if astrocytes-mediated response leads to increased abnormal levels of p50, or elevated levels of CCL2, then a dysregulated immune response could start, leading to an increased lymphocytes recruitment and widespread inflammatory response which are well-known signs of MS.

In order to further investigate which exposure from our selected set of closely related candidate genes are causally related MS we further apply a MVMR approach. MVMR allows to estimate the direct casual effect of each exposure in turn, i.e., each gene expression level, on MS when all the other exposures in the model are held constant, analogously to a multivariable regression. Furthermore, by including multiple exposures into a single model MVMR allows genetic variants to have pleiotropic effects on the exposure, i.e., *measured pleiotropy* (Rees et al., 2017; Zuber et al., 2020). If mediating effects between the gene expression levels are present, this method allows to identify the gene whose expression level has the greatest direct effect on the disease.

In our study we investigated the causal effect of genes related, in some way, to the same signaling pathway for which a mediated effect is likely.

The MVMR analysis identified in the cerebellum region three genes with the largest direct effect within our five selected genes in the NF-κB signaling pathway: *NKFB1*, *CCL2* and *TNFRSF1A*. As for these genes the direct effect in CRBL is different in magnitude from the total effect calculated in the univariable MR, we can hypothesized for these gene a mediation scenario in which the effect of *NFKB1* is in some way mediated by the effect of the other genes in the pathway, with an estimated direct casual OR = 2.11 with 95% CI [1.27;3.51] (more than double in magnitude compared to the total effect), the effect of *CCL2* is mediated by the effect of the other genes in the pathway, with a direct causal OR = 0.59 and 95% CI [0.42;0.83], and that the effect of *TNFRSF1A* is mediated by the effect of the other genes in the pathway, with a direct causal OR = 1.44 and 95% CI [1.08;1.92]. These results are also consistent with the biological evidence for which TNFRSF1A leads to the activation of different signaling pathways including NF-κB signaling pathway.

As regards MEDU, MVMR showed for *CCL2* a significant direct causal OR equal to 1.50 with 95% CI [1.04;2.17] greater in magnitude with respect to total effect suggesting also in this case a possible mediating effect of the other genes in the pathway.

In summary, our study aims at drawing a causal inference on expression of genes belonging to the same pathway, relevant for MS, both in term of total and direct causal effect. Direct and indirect effect in term of OR for the 5 genes in the cerebellum and medulla are summarized in Table 6.

**TABLE 6:** Direct and indirect effect (OR) of the 5 genes in the cerebellum and medulla brain region.

	*NFKB1*	*CCL2*	*CXCL10*	*MAPK14*	*TNFRSF1A*
**Cerebellum**					
Direct effect	2.11**	0.59**	1.06	0.64	1.44
Total effect	1.38**	0.97	1.19	1.01	1.04
**Medulla**					
Direct effect	0.98	1.50*	0.91	1.15	0.84
Total effect	1.02	1.31**	1.08	0.92	1.16

*p < 0.05, **p < 0.01.

The strengths of our analysis are: (*i.*) this is a rare application in which 5 exposures, are studied simultaneously, (*ii.*) MR allows to overcome potential confounding and reverse causation that may bias estimates in observational studies; (*iii.*) using data from two non-overlapping datasets allowed to avoid winner’s curse bias and bias toward the observational estimate, and to obtain more precise estimates than if individual-level data from one study were used. (*iv*) Finally, here we studied the causal effect of a lifelong exposure of gene expression levels with MS in the general European population, and, such an effect could not be studied with another study design including randomized controlled trial.

However, this study has some limitations: (*i.*) sample size is relatively small so that the study is underpowered to detect small but interesting causal effects; (*ii.*) the possibility that residual pleiotropy could bias the estimates; even if the main findings turned out to be robust in the sensitivity analyses, thereby decreasing the probability of bias due to pleiotropy; (*iii.*) like in most MR studies, it was not possible to directly assess whether canalization (compensatory feedback interactions) may have influenced our results. However, since canalization assumes that other physiological mechanisms may attenuate the effect of gene expression, such feedback interactions would tend to bias results toward the null. As a matter of fact, this study has generated results that are very far from the null. (*iv*) Limitations due to the use of post-mortem tissue may result from cell damages and DNA/RNA degradation; (*v.*) another limitation of our study could concern the difference between UKBEC and Sardinian data: UKBEC study includes individuals of European descent, while Sardinian dataset includes individuals from the founder population of Sardinia. This potential problem could arise if IVs found in the European population are not valid IVs in the Sardinian population, or if causal SNPs in linkage disequilibrium (LD) with the IV in UKBEC are not in LD with the causal SNP also in Sardinia population. However, as in canalization bias, this limitation tends to bias results toward the null, thus our findings could be at most underestimated. (*vi.*) as to statistical methodology we haven’t used novel methodology, but we applied the approaches suggested in literature to check the assumptions underlying an MR analysis and implemented a bootstrap approach, using a modified version of *mr_rucker_bootstrap* code, found in TwoSampleMR R package, where confidence intervals were estimated through the percentile method rather than assuming a Student’s t-distribution for the bootstrap distribution; (*vii.*) we reckon that the current methodology allowed us just to draw only general conclusion about the possible mediation effect between the different genes in the pathway without going into depth about the underlying biological mechanism.

In conclusion, our study supports the evidence that NF-κB pathway, already extensively studied in literature, plays a role not only in the peripheral immune system, but also in brain cells and in a causative way. This study shows that as expected genes belonging to the same pathway can mediate the effect of each other. This study provides a rationale to further investigate which role could play *CCL2* expression in MEDU, *NFKB1, TNFRSF1A* and *CCL2* expression in CRBL in the pathogenesis of MS, and eventually to understand why these tissues, are more susceptible to increased expression of these genes also taking into account that the brain displays remarkable cellular heterogeneity even within distinct brain regions. It is worthwhile noting that the strongest effect both total and direct has been found in CRBL that is commonly affected by MS, and whose signs significantly contribute to MS symptoms clinical disability (Wilkins, 2017).

## Data Availability Statement

The summary-level data for this study are available in the Supplementary Material.

## Ethics Statement

The study was approved by the ethics committee of the Azienda Sanitaria of Nuoro and was conducted in conformity with the 1954 Declaration of Helsinki in its currently applicable version and applicable Italian laws. All study participants gave written informed consent.

## Author Contributions

LB, CB, and TF conceived and supervised the study. TF, AN, and DG performed the statistical analysis and interpreted the results. AB, JM, and carried out the experiments. MP, AT, PB, and VS collected and stored samples and performed the clinical evaluation. TF, AN, and LB wrote the original draft. All authors participated in revising and editing the manuscript.

## Conflict of Interest

The authors declare that the research was conducted in the absence of any commercial or financial relationships that could be construed as a potential conflict of interest.
