# Supplementary figures and images for: Investigating the Causal Effect of Brain Expression of CCL2, NFKB1, MAPK14, TNFRSF1A, CXCL10 Genes on Multiple Sclerosis: A Two-Sample Mendelian Randomization Approach
Source: Front Bioeng Biotechnol. 2020 May 5;8:397. doi: 10.3389/fbioe.2020.00397 (PMC7216783; doi:10.3389/fbioe.2020.00397)

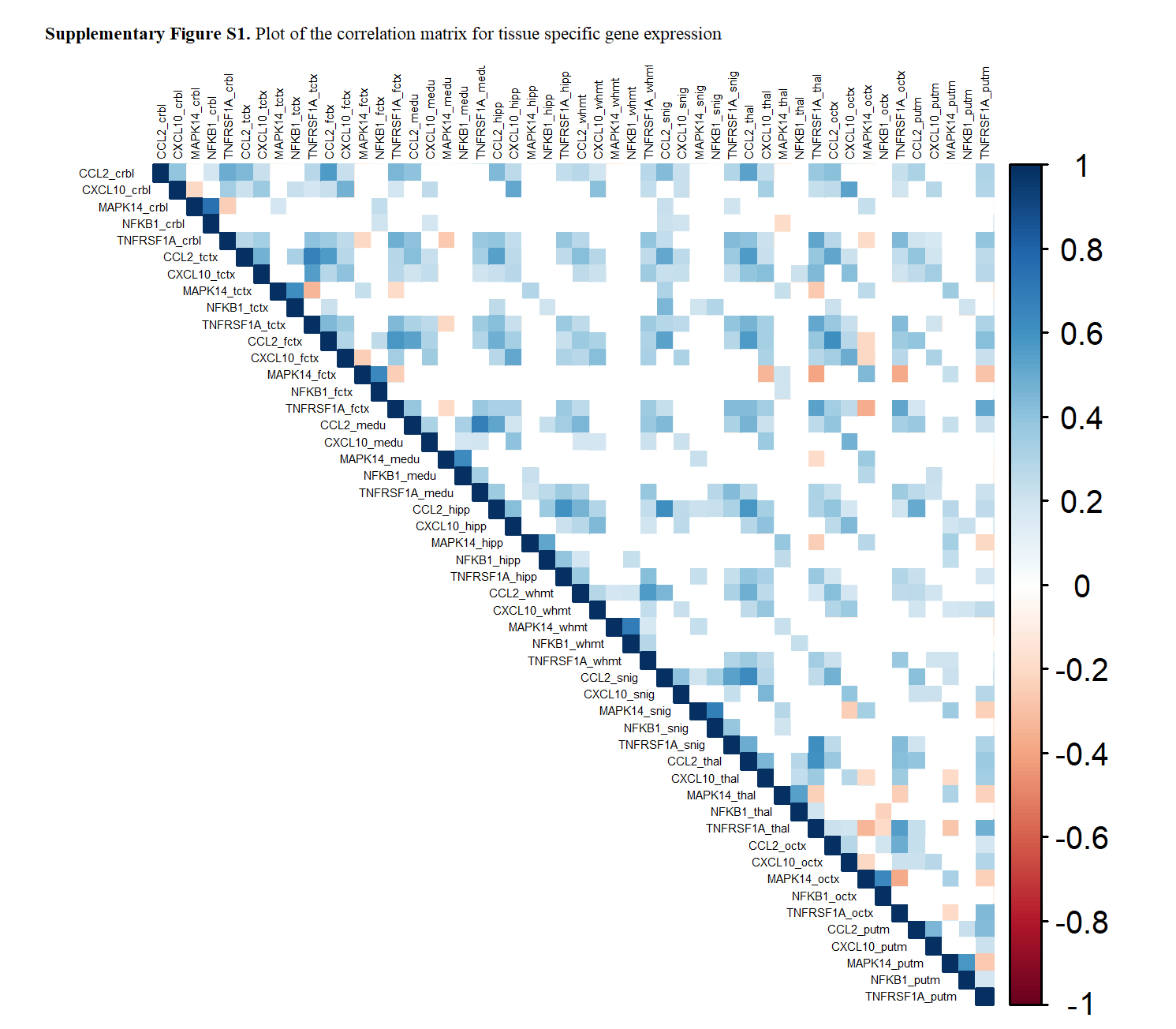

Supplement: Supplementary file 1 [file Image_1.tiff]
